# Distinct expression patterns of Notch ligands, Dll1 and Dll4, in normal and inflamed mice intestine

**DOI:** 10.7717/peerj.370

**Published:** 2014-05-01

**Authors:** Hiromichi Shimizu, Ryuichi Okamoto, Go Ito, Satoru Fujii, Toru Nakata, Kohei Suzuki, Tatsuro Murano, Tomohiro Mizutani, Kiichiro Tsuchiya, Tetsuya Nakamura, Katsuto Hozumi, Mamoru Watanabe

**Affiliations:** 1 Department of Gastroenterology and Hepatology, Tokyo Medical and Dental University, Tokyo, Japan; 2 Center for Stem Cell and Regenerative Medicine, Tokyo Medical and Dental University, Tokyo, Japan; 3 Department of Advanced Therapeutics for GI Diseases, Tokyo Medical and Dental University, Tokyo, Japan; 4 Department of Immunology, Tokai University School of Medicine, Isehara, Japan

**Keywords:** Dll1, Dll4, Hes1, ATOH1, Notch signaling, Intestinal epithelial cells, Colitis

## Abstract

Reports have suggested that the two Notch ligands, Dll1 and Dll4, are indispensable to maintain the homeostasis of the intestinal epithelium. However, within the intestinal epithelium, the precise distribution of the cells that express those ligands at the protein level remains largely unknown. Here, we show a series of immunohistochemical analysis through which we successfully identified mice intestinal epithelial cells (IECs) that endogenously express Dll1 or Dll4. Results showed that Dll1-positive (Dll1+ve) IECs reside exclusively within the crypt, whereas Dll4-positive (Dll4+ve) IECs can locate both in the crypt and in the villus of the small intestine. Also in the colon, Dll1+ve IECs resided at the lower part of the crypt, whereas Dll4+ve IECs resided at both upper and lower part of the crypt, including the surface epithelium. Both Dll1+ve and Dll4+ve IECs were ATOH1-positive, but Hes1-negative cells, and located adjacent to Hes1-positive cells within the crypts. A sub-population of both Dll1+ve and Dll4+ve IECs appeared to co-express Muc2, but rarely co-expressed other secretory lineage markers. However, as compared to Dll1+ve IECs, Dll4+ve IECs included larger number of Muc2-postive IECs, suggesting that Dll4 is more preferentially expressed by goblet cells. Also, we identified that Dll4 is expressed in the Paneth cells of the small intestine, whereas Dll1 and Dll4 is expressed in the c-kit-positive IECs of the colon, indicating that Dll1+ve and Dll4+ve IECs may contribute to constitute the intestinal stem cell niche. Compared to the normal colon, analysis of DSS-colitis showed that number of Dll1+ve IECs significantly decrease in the elongated crypts of the inflamed colonic mucosa. In sharp contrast, number of Dll4+ve IECs showed a significant increase in those crypts, which was accompanied by the increase in number of Hes1-positive IECs. Those Dll4+ve IECs were mostly found adjacent to the Hes1-positive IECs, suggesting that Dll4 may act as a major Notch ligand in the crypts of the inflamed colonic mucosa. Our results illustrate distinct expression patterns of Dll1 and Dll4 within the intestinal epithelium, and suggest that these two ligands may have different roles in normal and inflamed mucosa.

## Introduction

The intestinal epithelium is maintained by the rapid renewal of cells that are fueled by the stem cells residing at the lower part of the crypt (Crosnier, Stamataki & Lewis, 2006; Barker, 2013). Studies have identified that Lgr5-positive crypt base columnar cells definitely function as intestinal stem cells (Barker et al., 2007). These cells give rise to ATOH1-positive (ATOH1+ve) secretory-progenitor cells, or Hes1-positive (Hes1+ve) enterocyte-committed progenitor cells, which subsequently differentiate into one of the 5 types of mature cells. During such a process, various molecular signaling pathways including Notch plays distinct roles to maintain stem cell property (VanDussen et al., 2012; Clevers, 2013), and to organize proper proliferation and differentiation (Nakamura, Tsuchiya & Watanabe, 2007; Vooijs, Liu & Kopan, 2011).

Notch signaling is a pathway that is mediated by ligand–receptor interaction between neighboring cells (Artavanis-Tsakonas, Rand & Lake, 1999). So far, 4 receptors and 5 ligands have been identified in mammalians (Hori, Sen & Artavanis-Tsakonas, 2013). In the intestine, it has been shown that several components of the Notch pathway, including the 4 receptors and the 5 ligands are expressed during the developmental period and also during the adulthood (Schröder & Gossler, 2002). Studies in mice have shown that Notch1 and Notch2 are indispensable receptors to maintain the homeostasis of the intestinal epithelium (Riccio et al., 2008). Also, deletion of both Dll1 and Dll4 resulted in complete silencing of Notch activation within the intestinal epithelium, and thereby induced loss of stem-progenitor population, and a significant increase in number of secretory cells (Pellegrinet et al., 2011).

Recent report using Dll1-lacZ reporter mice have shown that Dll1 and Dll4 is expressed by the same population of intestinal epithelial cells (IECs) in the mice small intestine (Stamataki et al., 2011). Another report using *in situ* hybridization and a Dll1 knock-in mice showed that Dll1 is expressed in IECs at the +5 position, and those Dll1-positive (Dll1+ve) cells may exist as secretory lineage progenitor cells, and also as a back-up reservoir of stem cells (van Es et al., 2012). However, it remains unclear how the IECs that endogenously express the Dll1 protein locate within the crypt-villus unit, and possibly contribute to activate Notch signaling in their neighboring cells (van Den Brink, de Santa Barbara & Roberts, 2001). A recent report has shown that expression of Dll1 and Dll4 are directly regulated by the pro-secretory transcription factor, ATOH1, and may function as a key molecule to mediate lateral inhibition between equipotent progenitors (Kim et al., 2014). Nevertheless, the precise expression patterns of those ligands in the colon have never been described.

Here, we established an immunohistochemical method through which we can clearly identify cells that express Dll1 or Dll4 at the endogenous level. Results showed that Dll1 and Dll4 are expressed by a distinct subset of ATOH1+ve IECs that locates adjacent to Hes1+ve IECs in the small intestinal and colonic crypts. Moreover, Dll1+ve and Dll4+ve IECs appeared to change their dominance within the elongated crypts of the colitic mucosa, and thereby contribute to increase the number of Hes1+ve IECs. Thus, the present report clearly illustrates the differential expression patterns of Dll1 and Dll4 along the crypt-villus axis, under normal and colitic environment.

## Materials & Methods

### Mice

C57BL/6J mice at 6–8 weeks of age were purchased from Japan Clea (Tokyo, Japan). Lgr5- EGFP- ires- CreERT2 mice (Stock No. 008875) and R26R-lacZ mice (Stock No. 003309) were purchased from The Jackson Laboratory (Bar Harbor, Maine, USA). Dll1-floxed mice (Hozumi et al., 2004) and Dll4-floxed mice (Hozumi et al., 2008) have been previously described. To generate Lgr5- EGFP- ires- CreERT2/ Dll1^fl/fl^ mice or Lgr5- EGFP- ires- CreERT2/ Dll4^fl/fl^ mice, each floxed mice were crossed with Lgr5- EGFP- ires- CreERT2 mice. Mice carrying the R26R-LacZ allele served as control (Lgr5- EGFP- ires- CreERT2/ R26R-LacZ). Those mice were housed in the animal facility of Tokyo Medical and Dental University. All animal experiments were approved by the Institutional Animal Care and Use Committee of Tokyo Medical and Dental University (Approval Number 0140053A).

### Induction of Cre-mediated recombination

Induction of Cre-mediated gene recombination was induced by intraperitoneal injection of Tamoxifen (2 mg/body; SIGMA-ALDRICH, Missouri, USA) dissolved in corn oil for 5 consecutive days. Those mice were sacrificed at the indicated days after the first injection, and subjected to tissue analysis.

### Induction of colitis

Induction of colitis was performed as previously described (Okayasu et al., 1990; Okamoto et al., 2009; Yui et al., 2012). Briefly, mice were fed *ad libitum* with 3% Dextran sodium sulfate (DSS, Ensuiko, Yokohama, Japan) for five consecutive days, followed by distilled water for another five days. Mice fed with distilled water alone served as control. Mice were subjected to tissue analysis at the end of the experimental period (Day 10).

### Staining analysis of intestinal tissues

Immunostaining of mice intestinal tissues were done as previously described (Okamoto et al., 2009; Yui et al., 2012). Briefly, intestinal tissues were fixed in 4% parafolmaldehyde, and embedded in OCT compound (Sakura, Tokyo, Japan). 6 µm sections were prepared for staining. Primary antibodies used in the present study are summarized in Table 1. Microwave treatment (500 W, 10 min) in 10 mM citrate buffer was required for staining Dll1, Dll4, Hes1 and ATOH1. Tyramide signal amplification (Molecular Probes, California, USA) was used for immunofluorescent detection of Dll1, Dll4, Hes1 and ATOH1. Staining was visualized by secondary antibodies or Tyramide substrates conjugated with Alexa-594 or Alexa-488 (Molecular Probes, California, USA). Data were collected using a conventional epifluorecent microscope (BZ-8000 or BZ-X700, KEYENCE, Tokyo, Japan) or a confocal fluorescent microscope (FLUOVIEW FV10i; OLYMPUS, Tokyo, Japan). LacZ staining of the intestinal tissues were done as previously described (Barker et al., 2007).

**Table 1:** Primary antibodies used for immunohistochemical staining.

Antibody	Dilution	Supplier	Product no.	City	Country
Anti-Dll1	1:500	R&D systems	AF5026	Minneapolis	USA
Anti-Dll4	1:500	R&D systems	AF1389	Minneapolis	USA
Anti-Hes1	1:80000	A kind gift from Dr. T Sudo, Toray Industry		Kanagawa	Japan
Anti-ATOH1	1:200	A kind gift from Dr. JE Johnson, UT Southwestern		Dallas	USA
Anti-Ki-67	1:50	DAKO	TEC-3	Glostrup	Denmark
Anti-MUC2	1:100	Santa Cruz Biotechnology	Sc15334	Texas	USA
Anti-ChgA	1:1000	Diasorin	SP-1	Saluggia	Italy
Anti-DLCK1	1:100	Abgent	AP7219B	San Diego	USA
Anti-Lysozyme	1:1500	DAKO	EC3.2.1.17	Glostrup	Denmark
Anti-c-kit	1:100	R&D systems	AF1356	Minneapolis	USA
Anti-GFP	1:500	Nakalai Tesque	GF090R	Kyoto	Japan

### Quantification of the immunostaining

Number of positive staining cells was quantified by analyzing at least 100 crypts or villi that were randomly selected from 3 individual mice. Two independent investigators, who were not informed of the origin of the tissues or the targets of the staining, collected the data. Those data were statistically analyzed with Welch’s *t*-test.

## Results

### Dll1+ve and Dll4+ve IECs show distinct distribution pattern within the small intestinal and colonic epithelium

**Figure 1:**
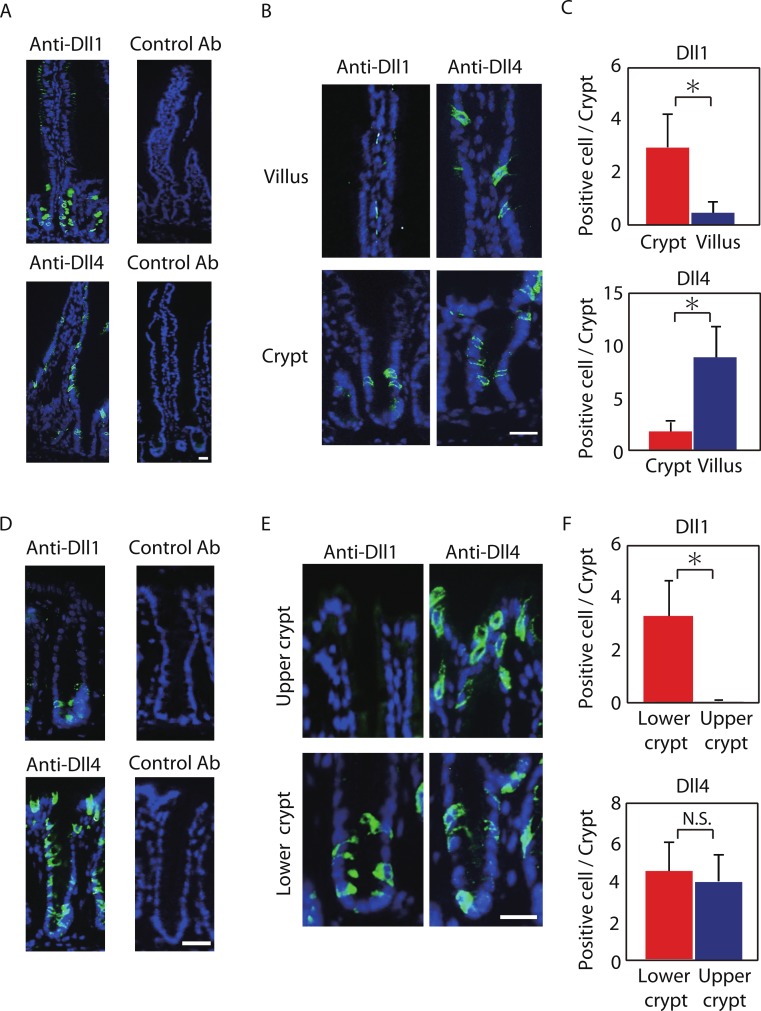
Dll1 and Dll4 are expressed in distinct patterns in the mice gastrointestinal epithelium. Immunohistochemistry of Dll1 and Dll4 were performed using mice small intestinal (A, B) and colonic tissues (D, E). Within the small intestinal epithelium, staining by anti-Dll1 antibody showed positive cells exclusively within the crypt (A, B), whereas staining by anti-Dll4 antibody showed positive cells both in the crypt and in the villi. Quantification of Dll1+ve and Dll4+ve intestinal epithelial cells (IECs) showed that Dll1+ve IECs are predominantly found within the crypt, whereas Dll4+ve IECs are found mostly in the villi (C). In the colonic epithelium, staining by anti-Dll1 antibody showed positive cells exclusively within the lower part of the crypt (D, E), whereas staining by anti-Dll4 antibody showed positive cells both in the lower- and upper part of the crypt, including the surface epithelium. Quantification of Dll1+ve and Dll4+ve IECs showed that Dll1+ve IECs are found exclusively within the lower half of the crypt, whereas Dll4+ve IECs are found both in the lower and upper region of the crypt at a comparable frequency (F). Negative staining of non-immunized isotype antibodies (Control Ab) confirmed the specific staining of Dll1 (A) and Dll4 (D). Scale bar represents 20 µm. Quantitative data are shown as mean ∓ SD of triplicate experiments (*n* = 3). ^∗^ indicates *P* < 0.05 as determined by Welch’s *t*-test. N.S. indicates not significant.

As previous studies have suggested that mRNA of Dll1 might be expressed within the mice intestinal epithelium (Schröder & Gossler, 2002; Crosnier et al., 2005; Stamataki et al., 2011; van Es et al., 2012), we sought to establish a method to identify cells that endogenously express Dll1 or Dll4 protein at their cell membrane, and thereby function as Notch signal-sending cells. By using a specific antibody for Dll1 or Dll4, we found that a positive staining can be yielded within the intestinal epithelium of the mice small intestine and the colon (Figs. 1A and 1D).

The specificity of those antibodies were validated and affirmed by conditional knockout of Dll1 or Dll4. By using mice carrying either the Dll1-floxed allele or the Dll4-floxed allele in addition to LGR5-EGFP-ires-CreERT2 allele, we induced genetic deletion of either Dll1 or Dll4 in LGR5-EGFP positive intestinal stem cells, and also in their progenies. In those knockout tissues, a clear abolishment of the positive staining in the progenies of EGFP-positive intestinal stem cells was confirmed in a gene-specific manner (Fig. S1).

Positive staining of both Dll1 and Dll4 showed a membranous pattern confirming that the staining demonstrates proteins that are bound to the membrane (Figs. 1B and 1E). However, the distribution of those cells along the crypt-villus axis showed a clear difference between Dll1-positive (Dll1+ve) and Dll4-positive (Dll4+ve) IECs. In the small intestine, Dll1+ve IECs resided exclusively within the crypts, but were never found in the villi (Fig. 1B). In sharp contrast, Dll4+ve IECs were found both in the villi and in the crypts. Quantitative analysis revealed that Dll1+ve IECs predominantly reside in the crypt, whereas Dll4+ve IECs mostly reside at the villus (Fig. 1C). Such a difference in distribution between Dll1+ve and Dll4+ve IECs were also found in the colon (Figs. 1D and 1E). In the colon, Dll1+ve cells were found predominantly at the lower half of the crypt, but were rarely found at the upper part of the crypt, including the surface epithelium (Fig. 1E). In contrast, Dll4+ve cells were found both in the upper and lower part of the crypt, and also at the surface epithelium. Quantitative analysis confirmed that Dll1+ve IECs reside predominantly in the lower part of the crypt, whereas Dll4+ve IECs reside both in the lower and upper part of the crypt (Fig. 1F). These results indicated that Dll1+ve and Dll4+ve IECs represent a distinct population of cells within the intestinal epithelium.

### Both Dll1+ve and Dll4+ve IECs are ATOH1-positive cells, and locate adjacent to Hes1-positive IECs in the small intestinal- and colonic-crypts

**Figure 2:**
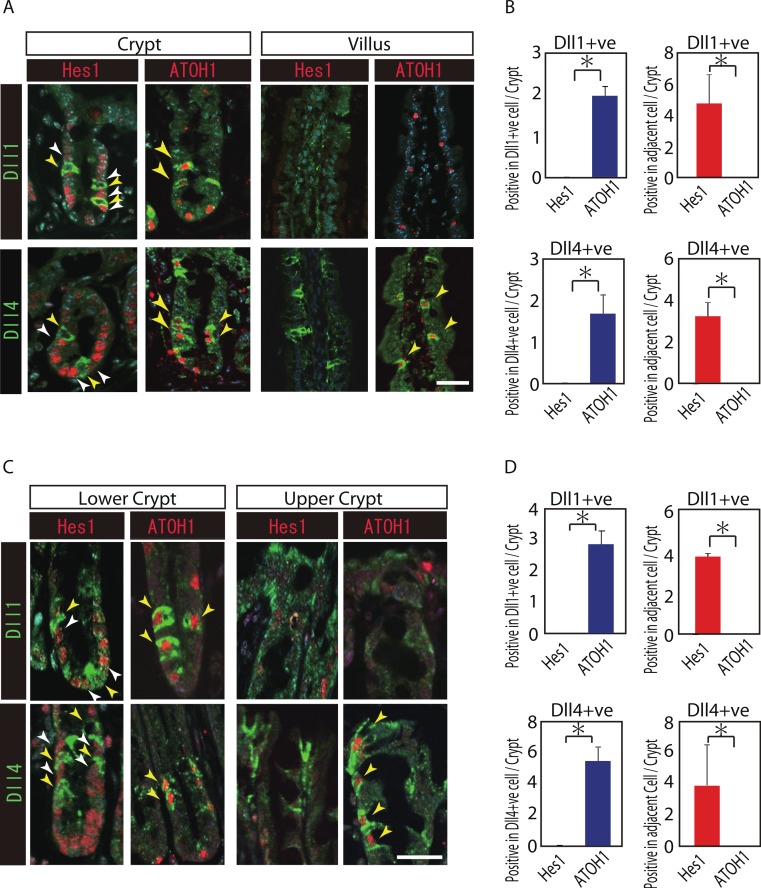
Both Dll1+ve and Dll4+ve IECs are ATOH1+ve IECs, which locate adjacent to Hes1+ve cells within the crypt. (A, C) Double immunostaining of Dll1 or Dll4 (green) with either Hes1 or ATOH1 (red) was performed in the mouse small intestinal (A) and colonic tissues (C). Both in the small intestinal and colonic epithelium, Dll1+ve IECs or Dll4+ve IECs itself were completely negative for Hes1 (yellow arrowhead), but instead, they were found adjacent to Hes1+ve IECs (white arrowhead) within the crypt. Those Dll1+ve IECs or Dll4+ve IECs were invariably positive for ATOH1 (yellow arrowhead). However, those Dll4+ve IECs that were residing at the small intestinal villi or in the colonic surface epithelium were never found adjacent to Hes1+ve IECs. Scale bar represents 20 µm. (B, D) Quantitative analysis of the immunostaining revealed that both Dll1+ve and Dll4+ve IECs are Hes1−ve/ ATOH1+ve cells that locate adjacent to Hes1+ve /ATOH1−ve IECs in the small intestinal (B) and colonic (D) crypts. Quantitative data are shown as mean ∓ SD of triplicate experiments (*n* = 3). ^∗^ indicates *P* < 0.05 as determined by Welch’s *t*-test. These data were acquired by confocal microscopy (FV10i).

Reports have suggested that Hes1 expression in IECs indicate Notch-activation and commitment to absorptive lineage cells, whereas ATOH1 expression in IECs conversely indicates lack of Notch activation and commitment to secretory lineage cells (van Den Brink, de Santa Barbara & Roberts, 2001; Vooijs, Liu & Kopan, 2011). To identify their potential to serve as Notch activating cells, and gain insight into their cell characteristics, we conducted a double immunostaining analysis of Dll1 or Dll4 with Hes1 or ATOH1. In the small intestinal crypts, both Dll1+ve and Dll4+ve cells were found adjacent to Hes1-positive (Hes1+ve) cells (Figs. 2A and 2B). In the villi, the adjacent cells of Dll4+ve cells were never found to be positive for Hes1. Also, both Dll1+ve and Dll4+ve IECs themselves were never found to be positive for Hes1. Instead, both Dll1+ve and Dll4+ve IECs were invariably positive for ATOH1 (Fig. 2B). Exactly the same expression patterns of Hes1 and ATOH1 within Dll1+ve and Dll4+ve IECs, or in their neighboring cells were found in the colonic crypts (Figs. 2C and 2D). A horizontal cross section of the crypt region further confirmed those expression patterns of Hes1 and ATOH1 in Dll1+ve and Dll4+ve IECs (Fig. S2). These findings clearly illustrate the definite site of lateral inhibition by ATOH1-positive (ATOH1+ve) crypt cells, through their expression of Dll1 or Dll4. In contrast, those Dll4+ve IECs that were found at the small intestinal villi or at the colonic surface epithelium appeared to co-express ATOH1, but were never found adjacent to Hes1+ve cells, indicating that those IECs may not function as Notch activating cells under normal conditions.

### Dll1+ve and Dll4+ve IECs represent distinct populations of secretory lineage cells in the small intestine and in the colon

**Figure 3:**
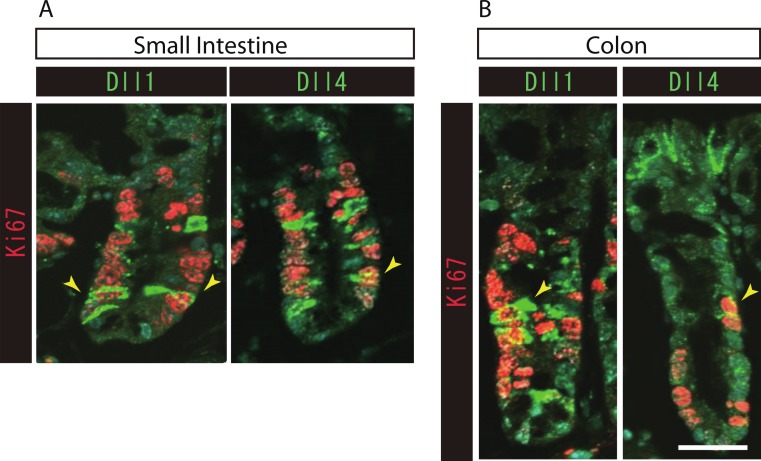
Both Dll1+ve and Dll4+ve IECs are mostly post-mitotic in the small intestine and in the colon. Double immunostaining of Dll1 or Dll4 (green) with the proliferation cell marker, Ki67 (red), shows that both Dll1+ve and Dll4+ve IECs are mostly post-mitotic in the small intestine (A) and in the colon (B). However, a small number of Dll1+ve or Dll4+ve IECs that co-express Ki67 can be found (yellow arrowhead). Scale bar represents 20 µm. These data were acquired by confocal microscopy (FV10i).

**Figure 4:**
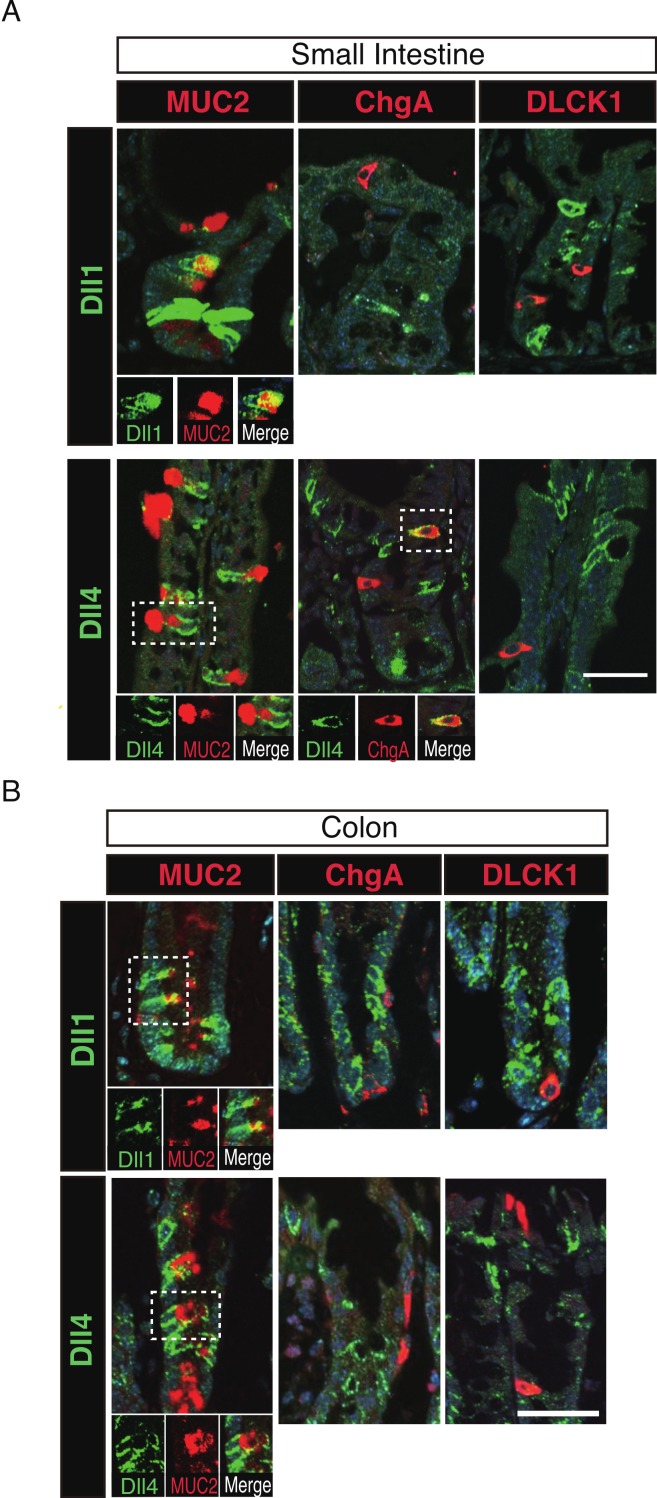
Dll1+ve and Dll4+ve IECs represent distinct populations of secretory lineage cells in the small intestine and in the colon. Double immunostaining of Dll1 or Dll4 with various secretory cell lineage markers were performed. (A) Immunostaining of Dll1 or Dll4 (green) with Muc2 (goblet cells, red), ChgA (Enteroendocrine cells, red) or DLCK1 (Tuft cells, red) using the small intestinal tissues shows that both Dll1+ve and Dll4+ve cells can co-express Muc2 (white square). Also, a small number of Dll4+ve cells that co-express ChgA were present (white square). However, Dll1+ve IECs were never found positive for ChgA or DLCK1. Also, Dll4+ve IECs were never found positive for DLCK1. Scale bar represents 20 µm. A magnified view of the area marked with a white square is shown in the lower panel. (B) The same immunostaining shown in (A) was performed using the colonic tissues. Both Dll1+ve and Dll4+ve colonic IECs can also co-express Muc2 (white square). However, both Dll1+ve and Dll4+ve IECs were never found positive for ChgA or DLCK1. Scale bar represents 20 µm. A magnified view of the area marked with a white square is shown in the lower panel. These data were acquired by confocal microscopy (FV10i).

To further identify the properties of Dll1+ve or Dll4+ve IECs, we further checked the co-expression of a proliferative cell marker, Ki67. Consistent with the former report (Stamataki et al., 2011), co-immunostaining with Ki67 showed that both Dll1+ve and Dll4+ve IECs are mostly negative for Ki67, indicating that they are post-mitotic in the small intestine (Fig. 3A) and in the colon (Fig. 3B). Quantitative analysis showed that 34.5% of Dll1+ve IECs and 19.3% of Dll4+ve IECs in the small intestine co-express Ki-67 (Table S1). Also, 22.6% of Dll1+ve IECs and 14.8% of Dll4+ve IECs in the colon co-expressed Ki-67. However, those values varied among individual crypts. As our former results suggested that both Dll1+ve and Dll4+ve IECs are ATOH1+ve cells that are committed to the secretory cell lineage, we analyzed the expression of secretory lineage differentiation markers in Dll1+ve or Dll4+ve IECs. In the small intestine, both Dll1+ve and Dll4+ve goblet cells were clearly present (Fig. 4A). Also, a few number of Dll4+ve enteroendocrine cells were found (Table S1). However, enteroendocrine cells that are positive for Dll1, or tuft cells that are positive for either Dll1 or Dll4, were never observed. In the colon, both Dll1+ve and Dll4+ve goblet cells were also clearly present (Fig. 4B). However, enteroendocrine cells or tuft cells that are positive for Dll1 or Dll4 were never observed. Quantitative analysis showed that increased number of Dll4+ve IECs co-express MUC2, compared to Dll1+ve IECs, both in the small intestine and in the colon (Table S1). Thus, the results indicated that both Dll1+ve and Dll4+ve IECs could acquire goblet cell phenotype, but rarely exhibit the phenotype of other secretory lineage cells. However, as compared to Dll1+ve IECs, Dll4+ve IECs appeared to include higher number of terminally differentiated goblet cells and can additionally acquire enteroendocrine cell phenotype, which confirmed the idea that Dll1+ve and Dll4+ve IECs represent distinct population of secretory lineage cells.

### Dll1+ve or Dll4+ve IECs constitute the intestinal stem cell niche

**Figure 5:**
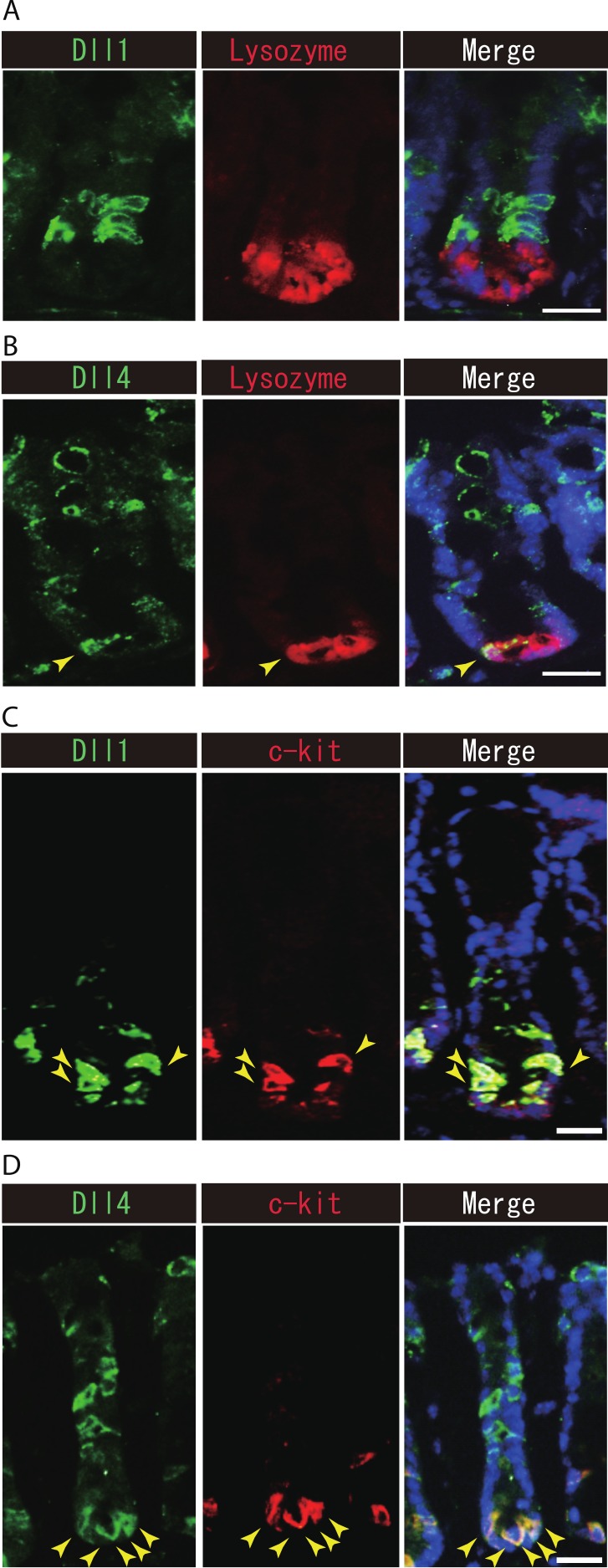
Dll1+ve or Dll4+ve IECs constitute the intestinal stem cell niche. Double immunostaining of Dll1 or Dll4 with a stem-cell niche cell marker, Lysozyme or c-kit, was performed in the mice small intestine and in the colon, respectively. (A) Immunostaining of Dll4 (green) with Lysozyme (red) shows that Dll4 is expressed in Paneth cells of the small intestine (yellow arrowhead). Scale bar represents 20 µm. (B) Double immunostaining of Dll1 (green) and c-kit (red) shows that Dll1+ve colonic IECs mostly co-express c-kit (yellow arrowhead). Also, double immunostaining of Dll4 (green) and c-kit (red) showed that a distinct population of Dll4+ve cells co-express c-kit (yellow arrowhead). Scale bar represents 20 µm.

Previous studies have suggested that Paneth cells express Dll1 or Dll4, and thereby constitute the key component of the small intestinal stem cell niche (Stamataki et al., 2011; Sato et al., 2012). However, endogenous protein expression of Dll1 or Dll4 in those stem cell niche cells has never been demonstrated. So far, our immunohistochemical analysis of small intestinal tissues failed to detect expression of Dll1 in any Paneth cell (Fig. 5A). However, a clear expression of Dll4 was found in a very rare population of Paneth cells (Fig. 5B). In the colon, it has been shown that c-kit positive cells take the place of Paneth cells, and constitute the colonic stem cell niche (Rothenberg et al., 2012). Double immunostaining with c-kit clearly showed that Dll1+ve IECs mostly overlap with the c-kit-positive cell population in the colon (Fig. 5C). Also, those Dll4+ve IECs that reside at the lower part of the colonic crypts clearly co-expressed c-kit (Fig. 5D). Quantitative analysis showed that almost all Dll1+ve IECs are c-kit positive, whereas around 70% of Dll4+ve IECs are c-kit positive in the colon (Table S1). Thus, our analysis showed that Dll1+ve IECs or Dll4+ve IECs surely reside as a component of the stem cell niche in small intestinal and colonic crypts.

On the other hand, our analysis of Lgr5-EGFP-ires-CreERT2 mice showed that Dll1+ve IECs or Dll4+ve IECs are mostly negative for LGR5-EGFP, suggesting that those cell populations do not include the LGR5-positive stem cell itself. However, we found a rare population of Dll1+ve or Dll4+ve IECs that co-express LGR5-EGFP, at the upper end of the stem cell region (Fig. S3). Those cells appeared to express EGFP at a relatively low level, and preferentially located at the +5 position. Thus these Dll1+EGFP+double positive cells, or Dll4+EGFP+double positive cells may represent the bi-potent secretory progenitor cells as described previously by van Es et al. (2012).

### Dll4+ve cells dominate the colonic crypts of the DSS-colitis mice

**Figure 6:**
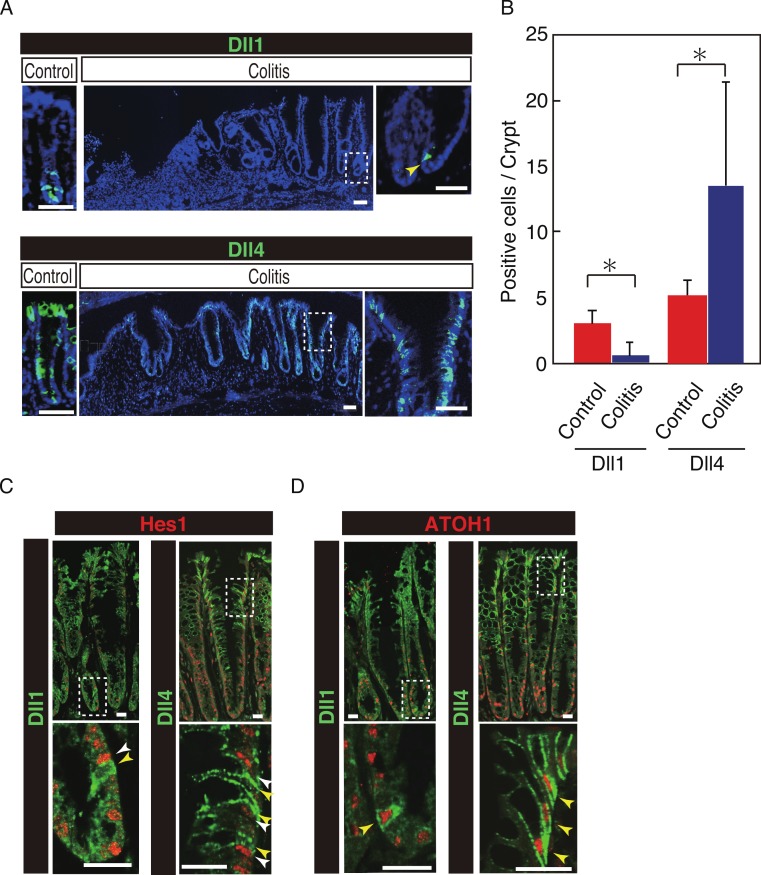
Dll4+ve cells dominate the colonic crypts of the DSS-colitis mice. Immunohistochemical analysis of colonic tissues that were prepared from DSS-colitis mice is shown. (A) Immunostaining of Dll1 and Dll4 using the inflamed colonic tissue of the DSS-colitis mice at day 10 (Colitis), or the corresponding tissue of the control mice (Control). In the inflamed colon, Dll1+ve IECs (green, upper panel) were rarely found within the crypts (yellow arrowhead). In sharp contrast, Dll4+ve IECs were frequently found, which dominated the entire crypt and also the surface epithelium. A magnified view of the area marked with a white square is shown in the right-end panel. Scale bar represents 50 µm. (B) Quantitative analysis of the staining shown in (A). Data shows a significant decrease in number of Dll1+ve IECs in the DSS-colitis mice (*n* = 3), compared to the control mice (*n* = 3). In contrast, a significant increase was found in number of Dll4+ve IECs in the DSS colitis mice, compared to control mice. Quantitative data are shown as mean ∓ SD. ^∗^ indicates *P* < 0.05 as determined by Welch’s *t*-test. (C) Double immunostaining of Hes1 (red) with either Dll1 or Dll4 (green). In the DSS-colitis mice, Hes1+ve cells were increased in number, and distributed up to the upper region of the elongated crypts. In such a crypt, a few Dll1+ve IECs (yellow arrowhead) were found at the lowest part of the crypt, which located adjacent to Hes1+ve IECs (white arrowhead). Dll4+ve IECs (yellow arrowhead) were frequently found in both lower and upper region of those crypts, which also located adjacent to Hes1+ve IECs (yellow arrowhead). Scale bar represents 20 µm. (D) Double immunostaining of ATOH1 (red) with either Dll1 or Dll4 (green). In the DSS-colitis mice, ATOH1+ve IECs were also increased in number, and distributed at both lower and upper region of the elongated crypts. In those crypts, Dll1+ve and Dll4+ve IECs were invariably positive for ATOH1 (yellow arrowhead). Scale bar represents 20 µm. The data shown in (C) and in (D) were acquired by confocal microscopy (FV10i).

Our previous study has shown that number of Hes1+ve cells significantly increase in the colitic mucosa of DSS-colitis mice, and also in ulcerative colitis patients, indicating that the canonical Notch pathway is activated in those cells (Okamoto et al., 2009; Zheng et al., 2011). However, the ligand that is responsible for such an increase in Notch activation remains uncertain. Therefore, we examined the expression of Dll1 and Dll4 in tissues of DSS-colitis mice. Analysis showed a surprising loss of Dll1 expression in the crypts of DSS-colitis mice (Fig. 6A). Conversely, a striking increase in the number of Dll4+ve IECs was observed in the crypts of those DSS-colitis mice, suggesting that a distinct regulation of Dll1 and Dll4 expression exists under the inflammatory environment. Quantitative analysis confirmed that number of Dll1+ve IECs significantly decrease, whereas that of Dll4+ve IECs significantly increase in DSS-colitis mice, compared to control mice (Fig. 6B). Double staining with Hes1 confirmed that a rare population of Dll1+ve IECs, as well as the dominant Dll4+ve IECs clearly located adjacent to Hes1+ve IECs within the elongated crypts of the DSS-colitis mice (Fig. 6C). Of note, those Hes1+ve IECs appearing at the mid-to-upper part of the colitic crypts were mostly accompanied adjacently by Dll4+ve IECs. Also, the analysis showed that both Dll1+ve and Dll4+ve IECs that were found in the DSS-colitis mice maintain their expression of ATOH1 (Fig. 6D). A horizontal cross section of the lower and upper crypt region further confirmed those expression patterns of Hes1 and ATOH1 in Dll4+ve IECs (Fig. S4). Thus, results indicated that the Dll1- or Dll4-ligand mediated Notch activation, as well as the lateral inhibition system, is maintained and present also in the inflamed colonic crypts.

## Discussion

In the present study, we showed that Dll1 and Dll4 are expressed in distinct patterns along the crypt-villus axis of the small intestine and the colon. The present distribution of Dll1 and Dll4 raised a possibility that ATOH1+ve secretory progenitor cells might express Dll1 at their earlier stage, and subsequently develop into Dll4+ve IECs as they become fully differentiated into goblet cells or enteroendocrine cells. Such a sequential expression of Dll1 and Dll4 during the process of lineage commitment has been formerly reported in the retina (Rocha et al., 2009). Former analysis by Stamataki et al., using Dll1-LacZ reporter mice, showed that Dll1+ve IECs overlap with Dll4+ve IECs in the mice small intestine (Schröder & Gossler, 2002). Such a complete overlap may due to the relatively longer half-life of the LacZ protein, compared to the endogenous Dll1 protein, and further indicate that Dll1+ve and Dll4+ve IECs develop along the same differentiation pathway, through which Dll1+ve IECs give rise to Dll4+ve IECs.

Also, the present difference in distribution of Dll1+ve and Dll4+ve IECs may provide an insight to the difference observed in the phenotype of Dll1- or Dll4-depletion in the intestinal epithelium. Conditional knockout studies have shown that depletion of Dll1 alone leads to a slight increase of secretory lineage cells, whereas depletion of Dll4 alone resulted in no distinguishable phenotype (Pellegrinet et al., 2011; Stamataki et al., 2011). Also, administration of the Dll4-neutralizing antibody had no effect on intestinal epithelial tissue homeostasis (Ridgway et al., 2006). Thus, Dll4+ve IECs may play a relatively limited role, compared to Dll1+ve IECs, in the normal intestinal epithelium. Given that a large number of Dll4+ve IECs were found at the villi or at the upper crypts, and did not appear to contribute to activation of Notch signaling in their adjacent cells, it may be quite easy to agree that depletion of Dll4 in such a cell population may result in only a minimal change. However, it remains possible that a difference in function may exist between Dll1 and Dll4, and therefore makes the Dll1+ve IECs functionally dominant over Dll4+ve IECs within the crypts. Further analysis of our Dll1 or Dll4 knockout mice (Fig. S1) may reveal how these ligands can compensate each other through the changes in their expression patterns.

Our present data clearly showed that colonic c-kit positive IECs could express Dll1 and Dll4 (Figs. 5C and 5D). Thus our data show that Dll1+ve and Dll4+ve IECs may constitute a part of the stem cell niche in the colonic crypts. In contrast, the low detection frequency of Dll1 or Dll4 expression in small intestinal Paneth cells might indicate that those cells are possible to activate Notch in adjacent stem cells at a relatively low level of Dll1 and Dll4 expression. Accordingly, studies using Dll1-LacZ mice have clearly demonstrated its reporter activity within Paneth cells (Stamataki et al., 2011).

We also found that the dominance of Dll1+ve or Dll4+ve IECs within the colonic crypt significantly shifts to Dll4+ve IECs in DSS-colitis mice. Those Dll4+ve IECs mostly located adjacent to Hes1+ve cells, thus indicating that they are contributing to activate Notch signaling in their neighboring IECs. In contrast, we frequently observed Dll4+ve IECs in the colonic crypts of the control mice, whose adjacent cells are clearly negative for Hes1. Thus Dll4+ve IECs in the normal intestinal crypts may serve as reserve cells that could immediately activate Notch signaling in adjacent cells upon tissue injury. The functional importance of Dll4+ve IECs in the colitic mucosa may be revealed by our forthcoming studies using intestinal-epithelial cell specific Dll4-knockout mice.

## Conclusions

Through our present results, we show that Dll1 and Dll4 are expressed by distinct population of ATOH1+ve cells in the small intestinal and colonic epithelium. Upon tissue injury, the dominance within the crypt is shifted to Dll4+ve IECs over Dll1+ve IECs. Therefore, our results suggest that Dll1+ve and Dll4+ve IECs may play different roles in the normal and inflamed colonic mucosa.

## Supplemental Information

10.7717/peerj.370/supp-1 Figure S1 Positive staining of Dll1 or Dll4 in IECs is completely abolished by conditional knockout of Dll1 or Dll4, respectively LGR5-EGFP-ires-CreERT2 mice carrying homozygous Dll1-floxed allele (LGR5-EGFP-ires-CreERT2/ Dll1^fl/fl^, *n* = 3), homozygous Dll4-floxed allele (LGR5-EGFP-ires-CreERT2/ Dll4^fl/fl^, *n* = 3), or a R26R-LacZ allele (LGR5-EGFP-ires-CreERT2/ R26R-LacZ, *n* = 3) were subjected to Cre-mediated gene recombination and analyzed 20 days after Tamoxifen-mediated induction (Day 1 indicates the day when the first Tamoxifen injection was performed). (A) LacZ staining (blue) of the small intestinal tissue derived from the LGR5-EGFP-ires-CreERT2/ R26R-LacZ mice confirms that at day 20, the whole crypt-villus unit can be completely replaced by the progeny of LGR5-EGFP+ve cells, in which Cre-mediated gene recombination had been induced. (B) Double immunostaining of Dll1 (green) and EGFP (red) in the small intestine (Day 20) is shown. Note that the positive signal of Dll1 is completely lost exclusively in the EGFP-positive crypt (highlighted by white dotted line) of LGR5-EGFP-ires-CreERT2/ Dll1^fl/fl^ mice. Such a loss of Dll1 positive signal was never found in mice carrying other genotypes. Scale bar represents 20 µm. (C) Double immunostaining of Dll4 (green) and EGFP (red) in the small intestine (Day 20) is shown. Note that the positive signal of Dll4 is completely lost exclusively in the EGFP-positive crypt (highlighted by white dotted line) and also in its extending villus (white arrow) of LGR5-EGFP-ires-CreERT2/ Dll4^fl/fl^ mice. Such a loss of Dll4 positive signal was never found in mice carrying other genotypes. Scale bar represents 20 µm.

10.7717/peerj.370/supp-2 Figure S2 Horizontal cross section analysis confirms that both Dll1+ve and Dll4+ve IECs are ATOH1+ve IECs, which locate adjacent to Hes1+ve cells within the crypt Double immunostaining of Dll1 or Dll4 (green) with either Hes1 or ATOH1 (red) was performed in horizontal cross sections of the mouse small intestinal and colonic crypts. Both in the small intestinal (A) and colonic (B) crypts, Dll1+ve IECs or Dll4+ve IECs itself were completely negative for Hes1 (yellow arrowhead, left panels). However, they were found adjacent to Hes1+ve IECs (white arrowhead, left panels) within the crypt. Those Dll1+ve IECs or Dll4+ve IECs were invariably positive for ATOH1 (yellow arrowhead, right panels). Scale bar represents 20 µm.

10.7717/peerj.370/supp-3 Table S1 Summary of proliferation and lineage marker expression in Dll1+ve or Dll4+ve IECs

10.7717/peerj.370/supp-4 Figure S3 A rare population of Dll1+ve and Dll4+ve IECs co-express EGFP in the small intestine of Lgr5- EGFP- ires- CreERT2 mice Double immunostaining of Dll1 or Dll4 (green) with the proliferation cell marker, Ki67 (red), shows that both Dll1+ve and Dll4 +ve IECs are mostly post-mitotic in the small intestine (A) and in the colon (B). However, a small number of Dll1+ve or Dll4 +ve IECs that co-express Ki67 can be found (yellow arrowhead). Scale bar represents 20 µm. These data were acquired by confocal microscopy (FV10i).

10.7717/peerj.370/supp-5 Figure S4 Horizontal cross section analysis confirms that both Dll1+ve and Dll4+ve IECs are ATOH1+ve IECs, and locate adjacent to Hes1+ve cells within the crypts of DSS-colitis mice (A) Double immunostaining of Hes1 (red) with either Dll1 or Dll4 (green) is shown. In the DSS-colitis mice, a few Dll1+ve IECs (yellow arrowhead) were found at the lower part of the crypt, which located adjacent to Hes1+ve cells (white arrowhead). Dll4+ve cells (yellow arrowhead) were frequently found in both lower and upper region of those crypts, which also located adjacent to Hes1+ve cells (white arrowhead). Scale bar represents 20 µm. (B) Double immunostaining of ATOH1 (red) with either Dll1 or Dll4 (green). In the DSS-colitis mice, ATOH1+ve cells distributed at both lower and upper region of the elongated crypts. Dll1+ve and Dll4+ve IECs were invariably positive for ATOH1 (yellow arrowhead). Scale bar represents 20 µm. Lower crypt section was defined as those found within 100 µm of vertical distance from the muscle layer, whereas upper crypt sections were defined as those found between 200 to 300 µm of vertical distance from the muscle layer. Note that the widely dilated crypt structure of the upper crypt sections is consistent with the vertical crypt structure shown in Figs. 6C and 6D.
