# Supplementary material for: Distinct expression patterns of Notch ligands, Dll1 and Dll4, in normal and inflamed mice intestine
Source: PeerJ. 2014 May 1;2:e370. doi: 10.7717/peerj.370 (PMC4017886; doi:10.7717/peerj.370)
Supplement: Table S1 [file peerj-02-370-s003.docx]

|  | **Small intestine** | | **Colon** | |
| --- | --- | --- | --- | --- |
|  | **% Positive in Dll1^+ve^ cells** | **% Positive in Dll4^+ve^ cells** | **% Positive in Dll1^+ve^ cells** | **% Positive in Dll4^+ve^ cells** |
| **Ki67*** | 34.5±9.6 | 19.3±6.0 | 22.6±10.5 | 14.8±8.4 |
| **MUC2**^#^ | 50.2±8.3 | 81.0±2.3 | 38.4±9.6 | 59.4±2.7 |
| **ChgA**^#^ | 0.0±0.0 | 3.1±2.4 | 0.0±0.0 | 0.0±0.0 |
| **DLCK1**^#^ | 0.0±0.0 | 0.0±0.0 | 0.0±0.0 | 0.0±0.0 |
| **Lysozyme^†^** | 0.0±0.0 | 0.46±0.26 | n.d. | n.d. |
| **c-kit^†^** | n.d. | n.d. | 99.8±0.4 | 70.8±3.8 |
| Data represents quantitative analysis of double-immunostainings shown in Fig. 3*, Fig. 4^#^ and Fig. 5**^†^**. Data are shown as Mean±SD. n.d. indicates not determined. | | | | |

Supplementary Table S1. Summary of proliferation and lineage marker expression in Dll1+ve or Dll4+ve IECs.
